# Saunders’s Question Compends

**Published:** 1903-06

**Authors:** 


Saunders's Question Compends. Essentials of Histology. By Louis Leroy, B.S., M.D , Professor of Histology and Pathology, Vanderbilt University, Medical and Dental Departments; Pathologist to the Nashville City Hospital, etc. Second edition, thoroughly revised and greatly enlarged. 16mo volume of 263 pages, with 92 illustrations. Philadelphia and London: W. B. Saunders & Co. 1902. [Cloth, $1.00 net.]

Saunders's question compends have doubtless filled a want in medical literature, as volume after volume of the series has passed into new editions. The volume on histology has been favorably received by the profession, inasmuch as the first edition was exhausted in a comparatively short time. The edition has been somewhat enlarged, especially the chapter on technic, which now is adequate to the needs of the student and general practitioner. Several new photomicrographs have been added and others replaced, making valuable additions to the little work.

Binding, paper and print are uniform with other volumes of the series and are commendable. W.C.K.

